# Supplementary material for: Translation and codon usage regulate Argonaute slicer activity to trigger small RNA biogenesis
Source: Nat Commun. 2021 Jun 9;12:3492. doi: 10.1038/s41467-021-23615-w (PMC8190271; doi:10.1038/s41467-021-23615-w)
Supplement: Supplementary file 1 — Supplementary Information [file 41467_2021_23615_MOESM1_ESM.pdf]

Translation and codon usage regulate Argonaute slicer activity to trigger small RNA biogenesis.

Singh *et al.,* 2021

Supplementary information

SUPPLEMENTARY FIGURES

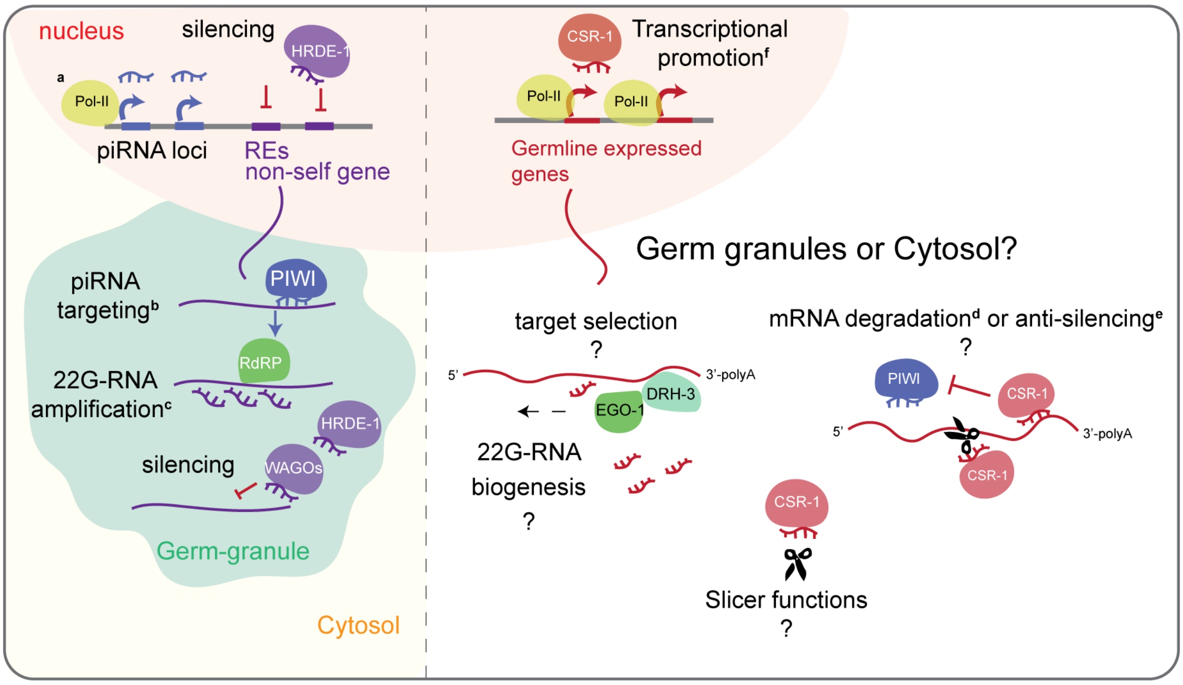

Supplementary Fig. 1: Current model for germline endogenous 22G-RNAs biogenesis and functions. a, PIWI interacting-RNAs (piRNAs, 21U-RNAs) are transcribed by RNA Polymerase II. **b-c,** piRNAs are then loaded by PIWI, which recruit an RNA-dependent RNA polymerase in germ granules to produce secondary antisense small RNAs (22G-RNAs) loaded by WAGOs, including nuclear Argonaute HRDE-1 and silence foreign transcripts like REs. CSR-1 loads 22G-RNAs synthesized by RdRP EGO-1. No trigger for CSR-1 22G-RNAs is known. Also, it is not clear where CSR-1 22G-RNAs are produced. CSR-1 possess a catalytic activity demonstrated in vitro. There is still a lack of clarity on the different proposed functions of CSR-1 and its slicer activity. **d,** Slicer activity is proposed to degrade mRNA either for maternal mRNA clearance in embryo or fine-tuning of mRNA levels to be delivered to oocytes. **e,** On the other hand, it is also proposed as an anti-silencer to PIWI mediated silencing of germline genes mRNAs and **f,** promote transcription of targets. However, it is not clear how CSR-1 targets are selected and how the RdRP EGO-1 mediated biogenesis of 22G-RNAs happens and where these activities occur- germ granule or cytosol?

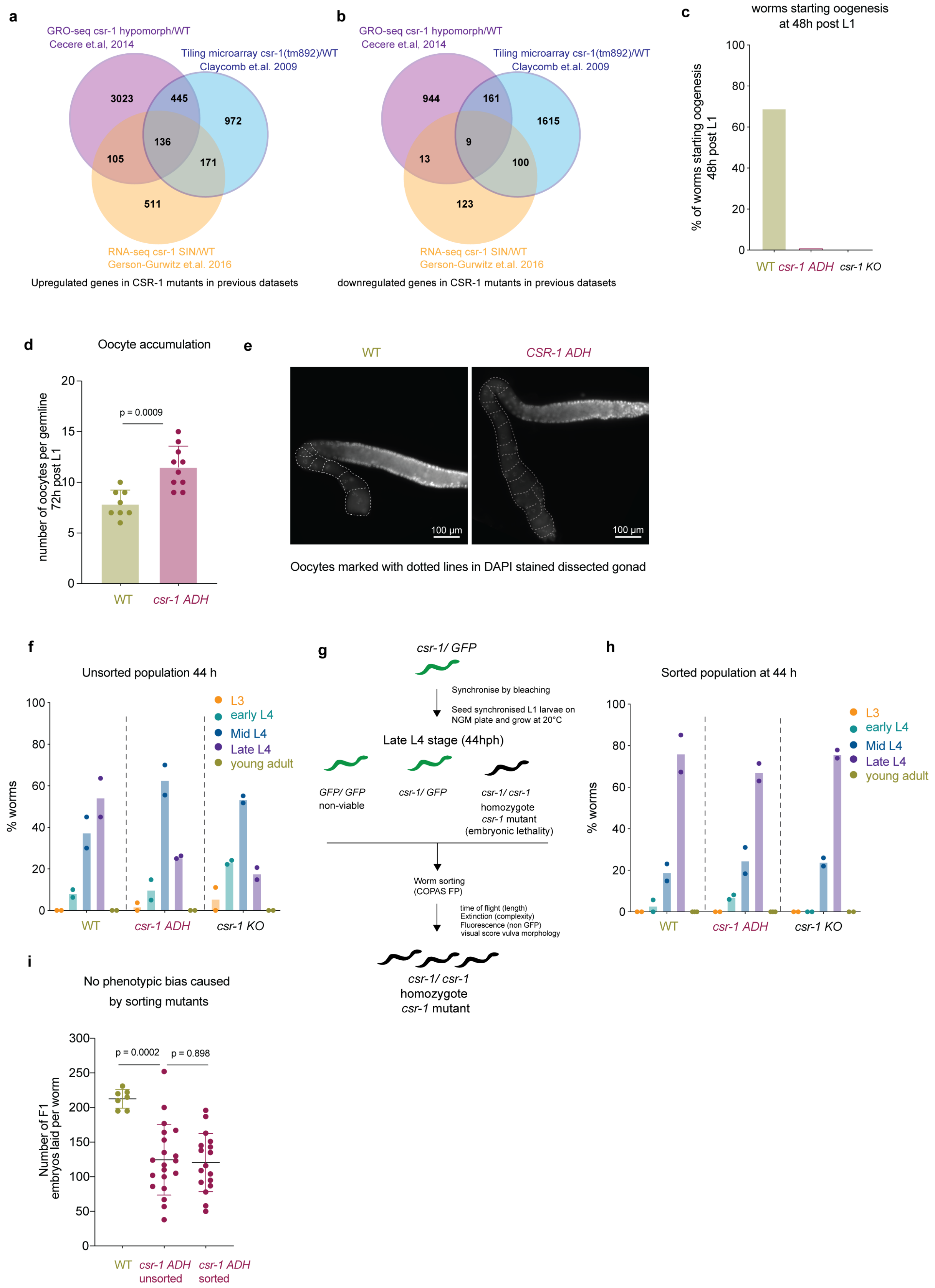

Supplementary Fig. 2: Phenotypic characterization of CSR-1 mutants.

a-b, Venn diagram showing overlap of upregulated **(a)** and downregulated **(b)** gene in different CSR-1 mutants in hermaphrodites investigated in the previous publications. No significant

overlap is observed. **c,** Comparison of the number of worms with oocytes at 48 h between WT strain, *csr-1* ADH and *csr-1* KO (n = 60 worms). **d,** Comparison of the number of oocytes present in the germline of adult worms at 72 h post-hatching between WT strain and *csr-1* ADH (n = 9 worms). **e,** Representative images of DAPI stained germlines of WT and *csr*-1 ADH showing increased accumulation of oocytes in *csr-1* ADH for data represented in (**d**). Oocytes are marked by white dashed lines (n = 9 worms). **f,** Distribution of WT*, csr-1* ADH and *csr-1* KO population in different larval stages after synchronization at 44 h post-hatching. Data are represented as mean (bar) for two biological replicates. **g,** Scheme for sorting synchronized WT or mutant worms using COPAS biosorter. **h,** Distribution of WT, *csr-1* ADH and *csr-1* KO population in different larval stages after sorting the synchronized worms at 44 h post-hatching. Data are represented as mean (bar) for two biological replicates used for sequencing in Figure 1 and Supplementary Figure 4. **i,** Brood size of *csr-1* ADH strain before and after sorting. Data are represented as mean ± s.d. Two-tailed P values were calculated using Mann–Whitney–Wilcoxon tests. (n=7 animals for WT, n=20 animals for *csr-1* ADH unsorted, n=17 animals for *csr-1* ADH sorted). Source data are provided as a Source Data file.

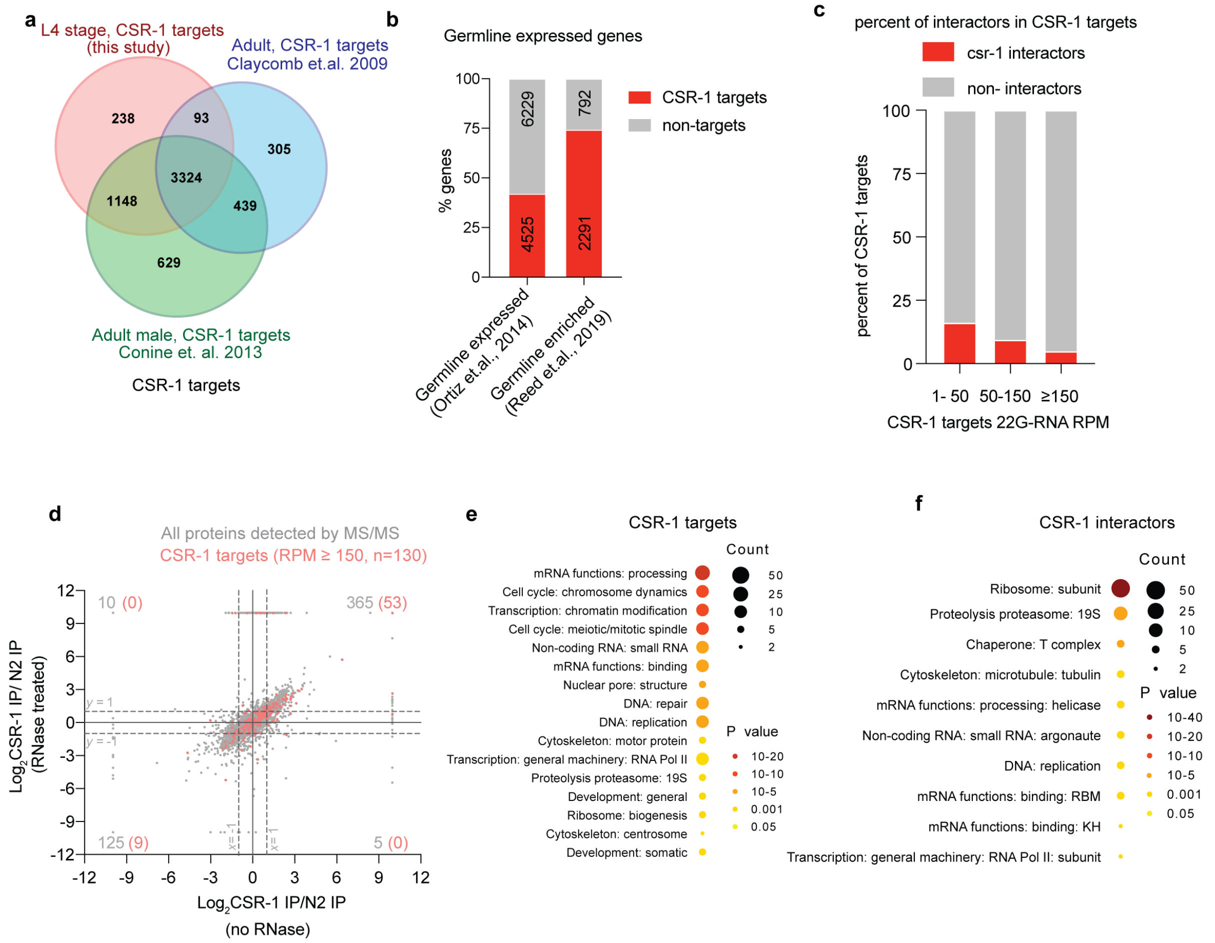

Supplementary Fig. 3: Phenotypic characterization of CSR-1 mutants.

a, Venn diagram showing overlap of CSR-1 targets identified by CSR-1 IP in the current study and previous publications. **b,** distribution of CSR-1 targets genes as a percent of the total for germline expressed and germline enriched genes. **c,** distribution of CSR-1 interactors as a percent of total for three categories of CSR-1 targets (with RPM of 1-50, 50-150 or ≥ 150 in the CSR-1 IP). **d,** Scatter plot comparing the $\log_2$ fold changes in CSR-1 interactors (IP-MS/MS) to control IPs performed in WT strain in the absence of RNase treatment (x-axis) to the IPs performed after RNase treatment. CSR-1 targets with 22G-RNA ≥150 RPM in the CSR-1 IP are highlighted. Dots in grey are all proteins detected in MS/MS analysis. Number in grey refers to all interactors with $\log_2$ fold change of ≥1 and p-value ≤ 0.05 for each quadrant. The number in parenthesis is CSR-1 targets with 22G-RNA ≥ 150 RPM. Enrichment factor for CSR-1 targets is 6.1 with $p < 1.6e^{-28}$. n = 4 biological replicates. **e-f,** gene categories enriched in CSR-1 targets (22G-RNA ≥ 150 RPM) **(e)** or CSR-1 interactors **(f).** Figures generated using WormCat. Source data are provided as a Source Data file.

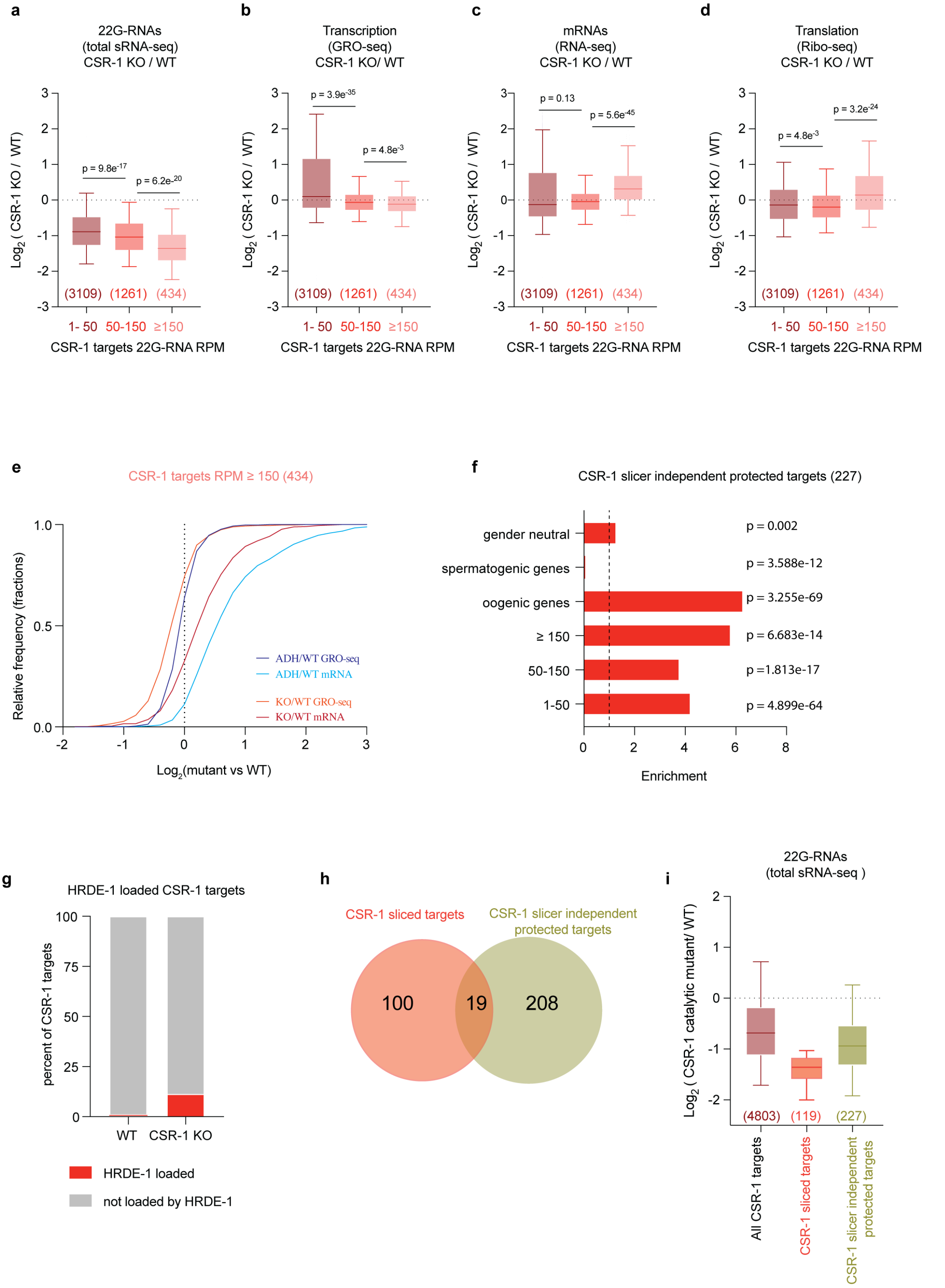

Supplementary Fig. 4: Gene expression analyses in *csr-1* KO mutant.

a-d, Box plots showing the $\log_2$ fold change of total 22G-RNAs (sRNA-seq) **(a);** nascent RNAs (GRO-seq) (**b**); or mRNAs (RNA-seq) **(c);** or mRNAs engaged in translation (Ribo-seq) (**d**),

in *csr-1* KO compared to WT strain. CSR-1 targets are categorized based on the abundance of the 22G-RNA in CSR-1 IP with 1-50 RPM, 50-150 RPM, or ≥ 150 RPM (see Supplementary Data 1 for gene lists). The line indicates the median value, the box indicates the first and third quartiles, and the whiskers indicate the 5th and 95th percentiles, excluding outliers. Two-tailed P values were calculated using Mann–Whitney–Wilcoxon tests. The sample size n (genes) is indicated in parentheses. Data is an average of 2 biological replicates. **e,** Cumulative frequency distribution for CSR-1 targets with RPM ≥ 150. The comparison shows GRO-seq ($p = 2e^{-11}$) and RNA-seq ($p = 9e^{-19}$) for *csr-1* KO for *csr-1* ADH compared to WT. Two-tailed P values were calculated using Mann–Whitney–Wilcoxon tests. The sample size n (genes) is indicated in parentheses. Data is an average of 2 biological replicates. **f,** Enrichment of CSR-1 slicer independent protected targets (Supplementary Data 1) in different CSR-1 targets categories based on 22G-RNA abundance, gender-neutral spermatogenic and oogenic genes. The dashed line at 1 indicates no enrichment. P values were calculated by Exact hypergeometric probability using the automated tool available at http://nemates.org/MA/progs/overlap_stats.html. **g,** distribution of CSR-1 target genes with 22G-RNA loaded by HRDE-1 as a percent of the total in WT or *csr-1* KO strain. **h,** Venn diagram showing overlap of CSR-1 sliced targets (targets with 2-fold upregulated mRNAs in *csr-1* ADH with 2-fold depleted 22G-RNAs, Fig. 1b and Supplementary Data 1) with CSR-1 protected targets (Supplementary Data 1) **i,** Box plots showing the log_2 fold change of total 22G-RNAs (sRNA-seq) in *csr-1* ADH compared to WT strain for all CSR-1 targets, CSR-1 sliced targets and CSR-1 protected targets. The line indicates the median value, the box indicates the first and third quartiles, and the whiskers indicate the 5th and 95th percentiles, excluding outliers. Two-tailed P values were calculated using Mann–Whitney–Wilcoxon tests. The sample size n (genes) is indicated in parentheses. Data is an average of 2 biological replicates. Source data are provided as a Source Data file.

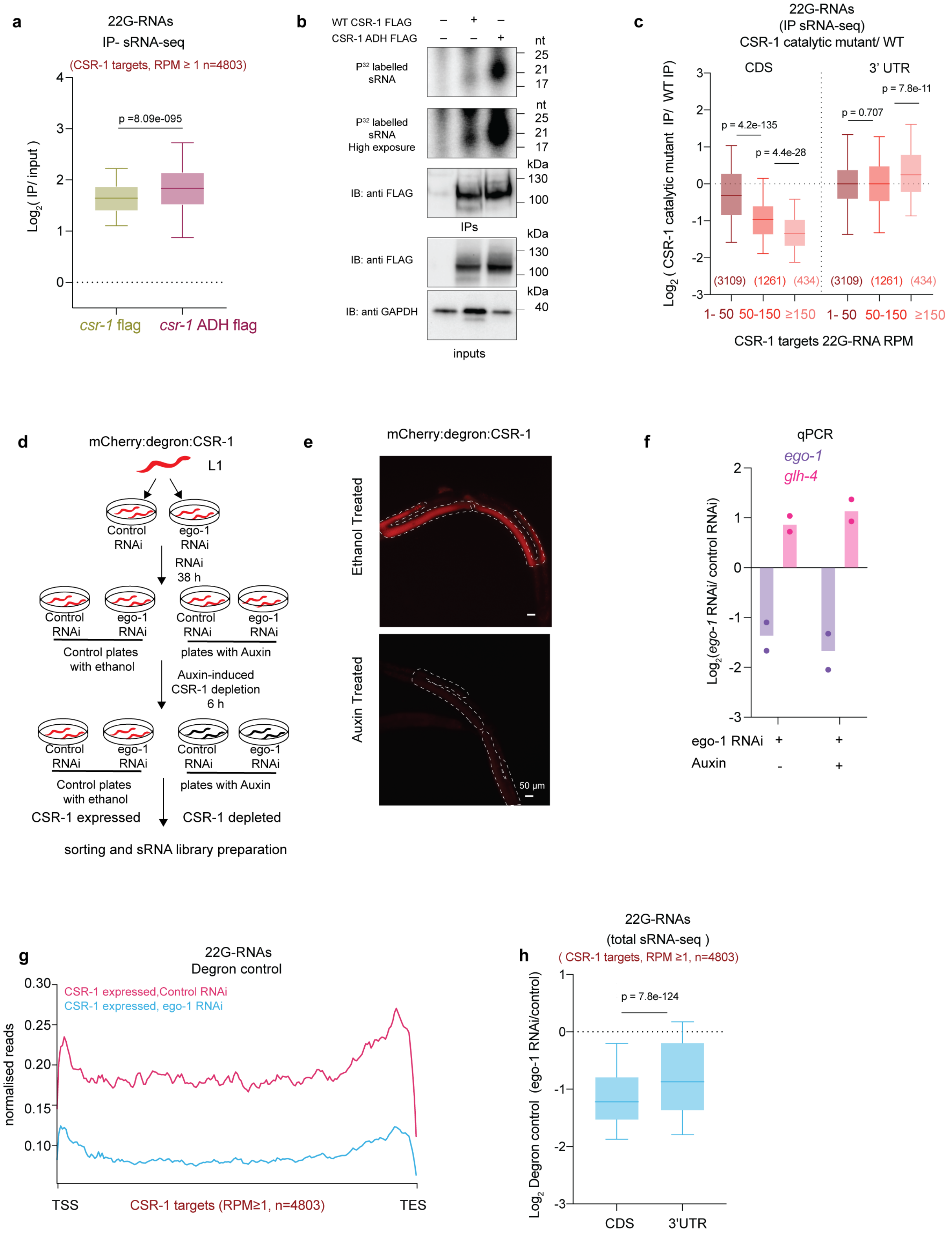

Supplementary Fig. 5: CSR-1 catalytic activity regulates the biogenesis of 22G-RNAs.

a, Box plot showing $\log_2$ fold change in 22G-RNAs in CSR-1 ADH and WT CSR-1 IPs compared to input for all CSR-1 targets. The sample size n (genes) is indicated in parentheses. Data is an average of 2 biological replicates. **b,** Autoradiograph showing the P^{32} radiolabeled

co-purifying sRNAs from control, WT FLAG::CSR-1 and FLAG::CSR-1 ADH immunoprecipitates. Immunoblot for WT CSR-1 and CSR-1 ADH using anti-FLAG antibody in the IPs and inputs. GAPDH was used as a loading control. The experiment was reproduced thrice. **c,** Box plot showing $\log_2$ fold change in 22G-RNAs in IPs of CSR-1 ADH compared to WT CSR-1 on CDS and 3'UTR. The distribution for the 22G-RNA in CSR-1 IP for CSR-1 targets with 1-50 RPM, 50-150 RPM, or ≥ 150 RPM (see Supplementary Data 1 for gene lists). The sample size n (genes) is indicated in parentheses. Data is an average of 2 biological replicates. **d,** Experimental scheme and **e,** fluorescent images of live animals expressing Degron::mCherry::3xFLAG::HA:: CSR-1 used for CSR-1 depletion experiments for Fig. 2g-i, and Supplementary Fig. 5f-h. Auxin treatment completely depletes CSR-1 in the germline. The image is representative of at least ten individual germlines from two biological replicates. **f,** Log_2 fold change in expression of RdRP *ego-1* mRNA and *glh-4* (CSR-1 target misregulated upon CSR-1 depletion) upon *ego-1* RNAi compared to control RNAi by qPCR for two biological replicates used for sequencing 22G-RNAs in Fig. 2g-i and Supplementary Fig. 5g-h. Data shows mean. **g,** Metaprofile analysis as in (Fig. 2g) showing the distribution of normalized total 22G-RNA reads (RPM) across CSR-1 targets (22G-RNA ≥1 RPM) upon *ego-1* RNAi and Control RNAi treated in degron control. **h,** Box-plot as in (Fig. 2h) showing the $\log_2$ fold change in the amount of 22G-RNA generated from CDS and 3' UTR of CSR-1 targets (22G-RNA ≥1 RPM) in *ego-1* RNAi compared to control RNAi treated in degron control. Data is an average of 2 biological replicates. For all the box plots, the line indicates the median value, the box indicates the first and third quartiles, and the whiskers indicate the 5th and 95th percentiles, excluding outliers. Two-tailed P values were calculated using Mann–Whitney–Wilcoxon tests. Source data are provided as a Source Data file.

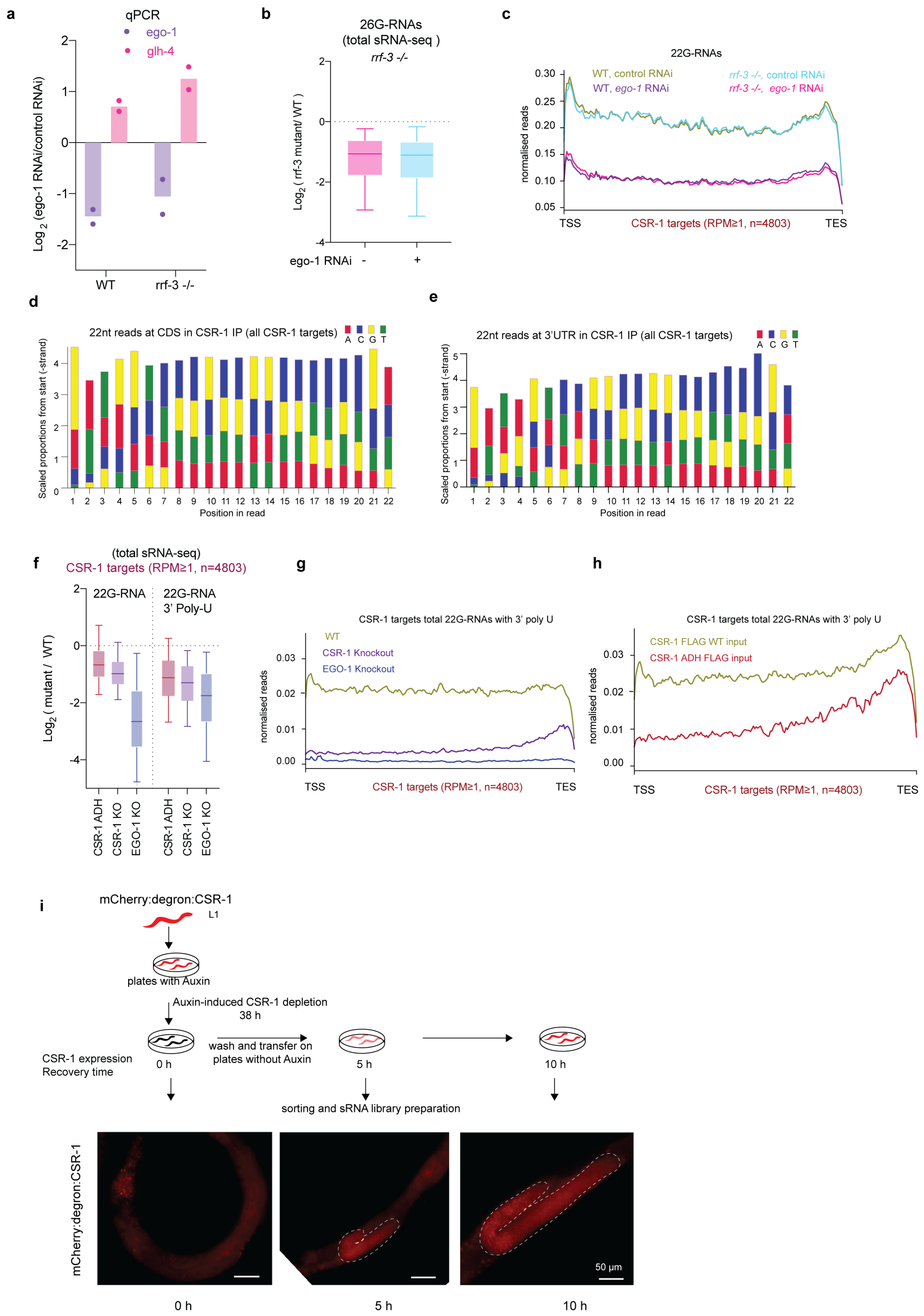

Supplementary Fig. 6: Characterization of CSR-1 22G-RNAs.

a, Log_2 fold change in expression of RdRP *ego-1* mRNA and *glh-4* (CSR-1 target misregulated upon CSR-1 depletion) upon *ego-1* RNAi compared to control RNAi by qPCR in WT and *rrf-1 -/-,* for two biological replicates used for sequencing 22G-RNAs in Supplementary Fig. 6b-

c. Data shows mean. **b,** Box plot showing $\log_2$ fold change in total 26G-RNAs in *rrf-3 -/-* compared to WT in presence and absence of *ego-1* RNAi. The line indicates the median value, the box indicates the first and third quartiles, and the whiskers indicate the 5th and 95th percentiles, excluding outliers. Two-tailed P values were calculated using Mann–Whitney–Wilcoxon tests. Data is an average of 2 biological replicates. **c,** Metaprofile analysis showing the distribution of normalized 22G-RNA (sRNA-seq) reads (RPM) along all CSR-1 targets (≥1 RPM, n= 4803) in WT or *rrf-3 -/-* in control and *ego-1* RNAi. TSS indicates the transcriptional start site, TES indicates the transcriptional termination site. The average of two biological replicates is shown. **d-e,** scaled proportions of nucleotide composition on each position of reads for sRNAs from all CSR-1 targets (n=4803) co-purifying with CSR-1 at CDS (coding sequence) **(d)** and 3' UTR **(e)**. **f,** Box plot showing $\log_2$ fold change in total 22G-RNAs of 22G RNAs with 3'poly U for *csr-1* ADH, *csr-1* KO and *ego-1* KO compared to WT. The line indicates the median value, the box indicates the first and third quartiles, and the whiskers indicate the 5th and 95th percentiles, excluding outliers. Two-tailed P values were calculated using Mann–Whitney–Wilcoxon tests. The sample size n (genes) is indicated in parentheses. Data is the mean of 2 biological replicates. **g-h,** Metaprofile analysis showing the distribution of normalized 22G-RNA with 3'poly U reads along all CSR-1 targets (≥1 RPM, n= 4803) in WT, *csr-1* KO and *ego-1* KO **(g)** and WT *csr-1* and *csr-1* ADH. TSS indicates the transcriptional start site, TES indicates the transcriptional termination site. The average of two biological replicates is shown. **i,** Experimental scheme and fluorescent images of live animals expressing Degron::mCherry::3xFLAG::HA::CSR-1 used for CSR-1 expression recovery for 0, 5 or 10 h after depletion for 38 h experiments for Fig. 2j-k. Images are representative of at least 10 individual germlines from two biological replicates. Source data are provided as a Source Data file.

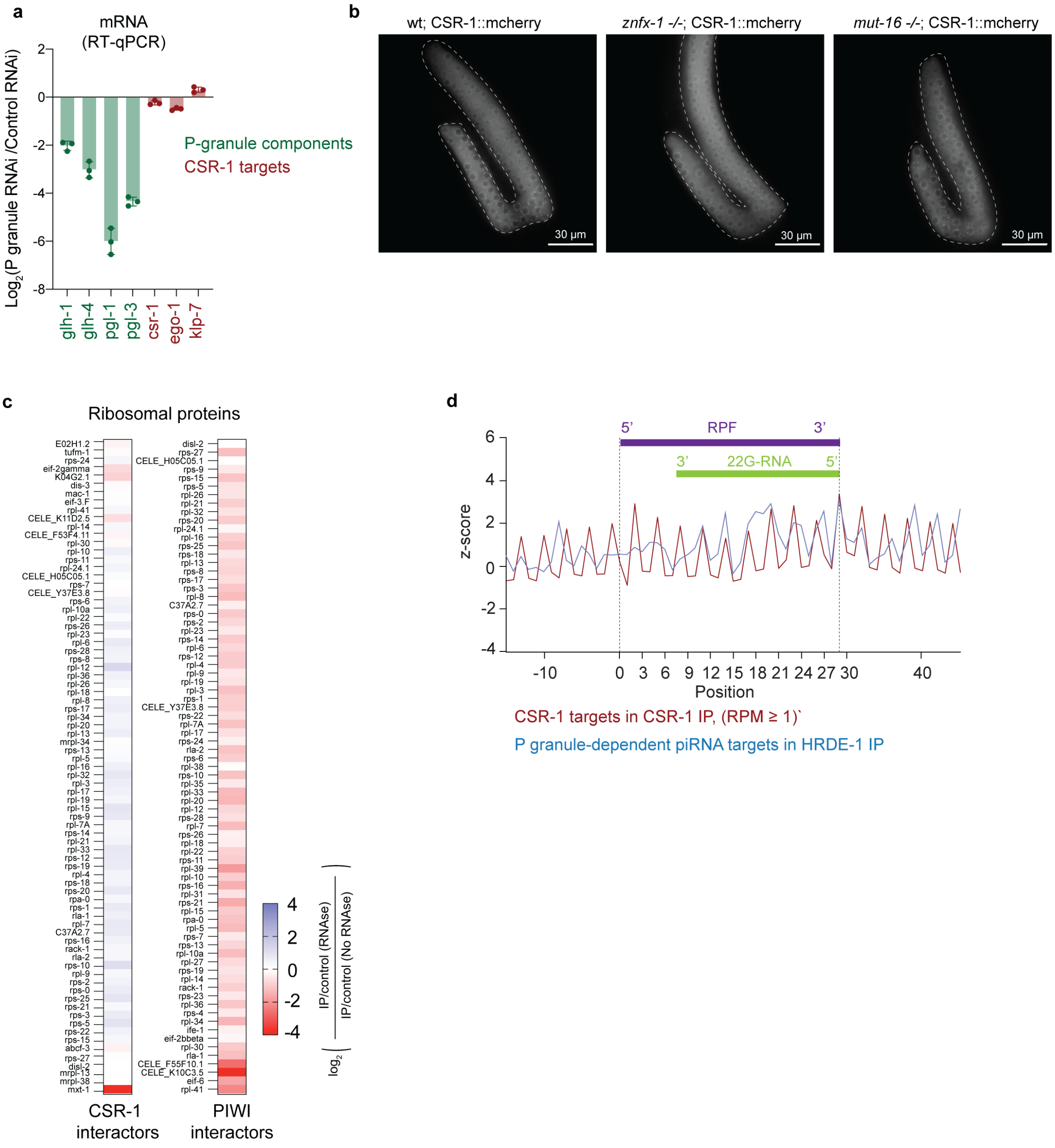

Supplementary Fig. 7: CSR-1 localization in P granule vs cytosol

a, $\log_2$ fold change in expression of *glh-1, glh-4, pgl-1* and *pgl-3* (germ granule components) and *csr-1, ego-1* and *klp-7* (CSR-1 targets) upon P granule RNAi compared to control RNAi by RT-qPCR for three biological replicates. Data is represented as mean ± SD. **b,** Live fluorescent images showing localization of mCherry:CSR-1 in WT, *znfx-1* -/- and *mut-16 -/-*. CSR-1 is localized in the P granule as well as cytosol. At least five individual germlines were imaged. **c,** heat map comparing $\log_2$ fold change for ribosomal interactors of CSR-1 and PIWI in the presence of RNase treatment compared to no RNase treatment. Ribosomal interactors are lost in PIWI and not in CSR-1 upon RNase treatment (Supplementary Data 3 and 5). n= 4

biological replicates. **d,** Plot showing the z-score for read density for the of 5' terminus of 22G-RNAs for CSR-1 targets (RPM≥1) in CSR-1 IP and P granule dependent piRNA targets in HRDE-1 IP (gene list in Supplementary Data 1) relative to the start of 29-nt long Ribosomal protected fragments (RPF) (Related to Fig. 4e). Data is representative of two biological replicates. Source data are provided as a Source Data file.

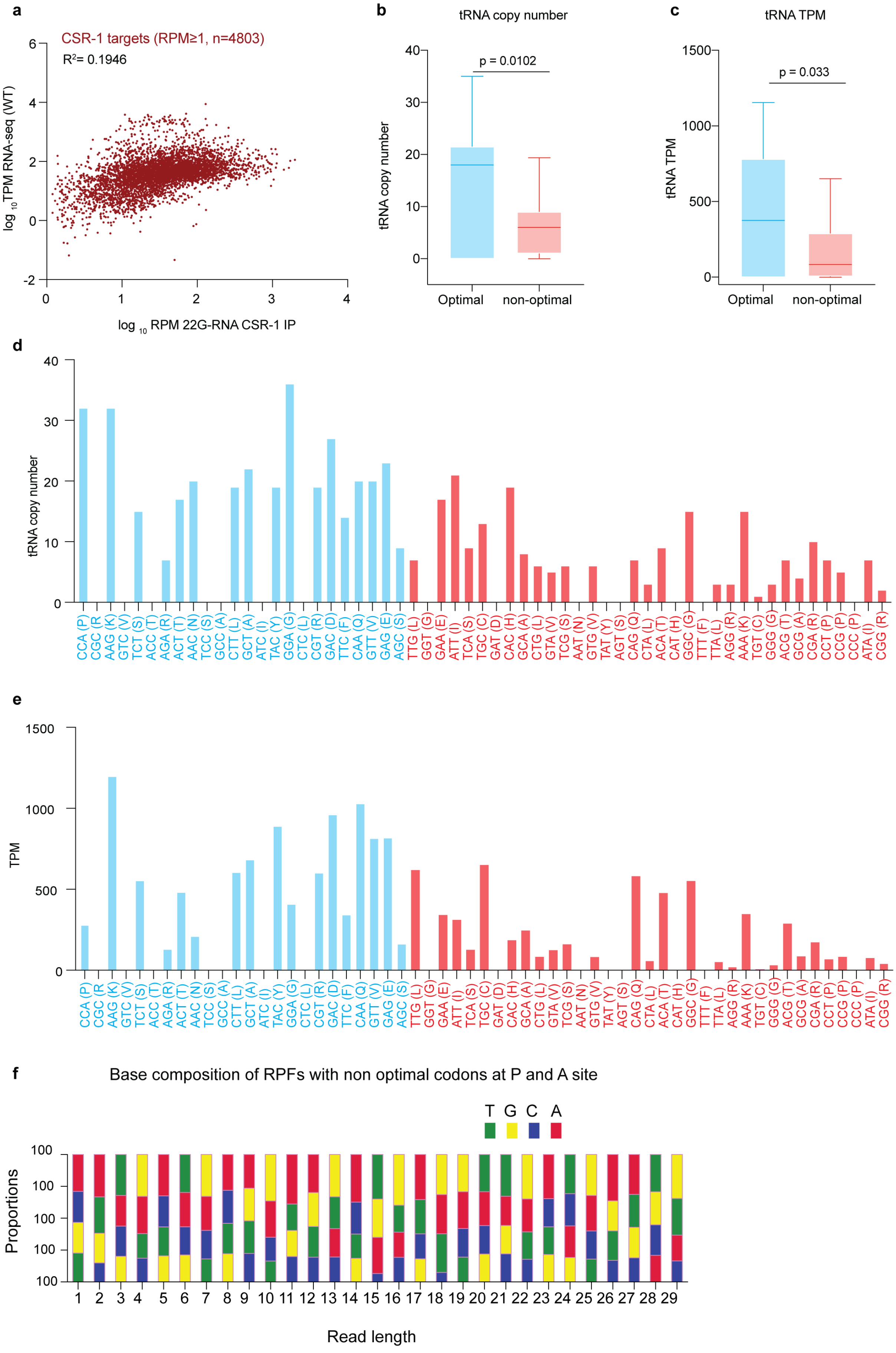

a
CSR-1 targets (RPM≥1, n=4803)
R²= 0.1946
log 10 TPM RNA-seq (WT)
log 10 RPM 22G-RNA CSR-1 IP
b
tRNA copy number
p = 0.0102
tRNA copy number
Optimal
non-optimal
c
tRNA TPM
p = 0.033
tRNA TPM
Optimal
non-optimal
d
tRNA copy number
CCA (P) CGC (R AAG (K) GTC (V) TCT (S) ACC (T) AGA (R) ACT (T) AAC (N) TCC (S) GCC (A) CTT (L) GCT (A) ATC (I) TAC (Y) GGA (G) CTC (L) CGT (R) GAC (D) TTC (F) CAA (Q) GTT (V) GAG (E) AGC (S) TTG (L) GGT (G) GAA (E) ATT (I) TCA (S) TGC (C) GAT (D) CAC (H) GCA (A) CTG (L) GTA (V) TCG (S) AAT (N) GTG (V) TAT (Y) AGT (S) CAG (Q) CTA (L) ACA (T) CAT (H) GGC (G) TTT (F) TTA (L) AGG (R) AAA (K) TGT (C) GGG (G) ACG (T) GCG (A) CGA (R) CCT (P) CCG (P) CCC (P) ATA (I) CGG (R)
e
TPM
CCA (P) CGC (R AAG (K) GTC (V) TCT (S) ACC (T) AGA (R) ACT (T) AAC (N) TCC (S) GCC (A) CTT (L) GCT (A) ATC (I) TAC (Y) GGA (G) CTC (L) CGT (R) GAC (D) TTC (F) CAA (Q) GTT (V) GAG (E) AGC (S) TTG (L) GGT (G) GAA (E) ATT (I) TCA (S) TGC (C) GAT (D) CAC (H) GCA (A) CTG (L) GTA (V) TCG (S) AAT (N) GTG (V) TAT (Y) AGT (S) CAG (Q) CTA (L) ACA (T) CAT (H) GGC (G) TTT (F) TTA (L) AGG (R) AAA (K) TGT (C) GGG (G) ACG (T) GCG (A) CGA (R) CCT (P) CCG (P) CCC (P) ATA (I) CGG (R)
f
Base composition of RPFs with non optimal codons at P and A site
T G C A
Proportions
Read length

Supplementary Fig. 8: tRNA copy number and expression for optimal and non-optimal codons.

a, Scatter plot showing the comparison of $\log_{10}$ RPM of 22G-RNAs from CSR-IP and $\log_{10}$ TPM of mRNAs in WT for all CSR-1 targets. The sample size n (genes) is indicated in parentheses. Data is an average of 2 biological replicates. **b-c,** box plot showing the copy numbers for tRNAs for optimal or non-optimal codons **(b)**, and the TPMs for tRNAs from the GRO-seq dataset for WT strain at the late l4 larval stage (44h) for optimal or non-optimal codons **(c),** as in Fig. 5, without adjusting for absent tRNAs. The line indicates the median value, the box indicates the first and third quartiles, and the whiskers indicate the 5th and 95th percentiles, excluding outliers. Two-tailed P values were calculated using Mann–Whitney–Wilcoxon tests. Data is an average of 2 biological replicates. **d,** Plot showing the copy number of tRNAs in the genome for each codon. **e,** Plot shows the tRNA pool's availability corresponding to each codon (TPM for tRNAs from GRO-seq, n = two biological replicates). **f,** Proportions of nucleotide composition for each position of RPF with A and P site occupied by non-optimal codons. Source data are provided as a Source Data file.

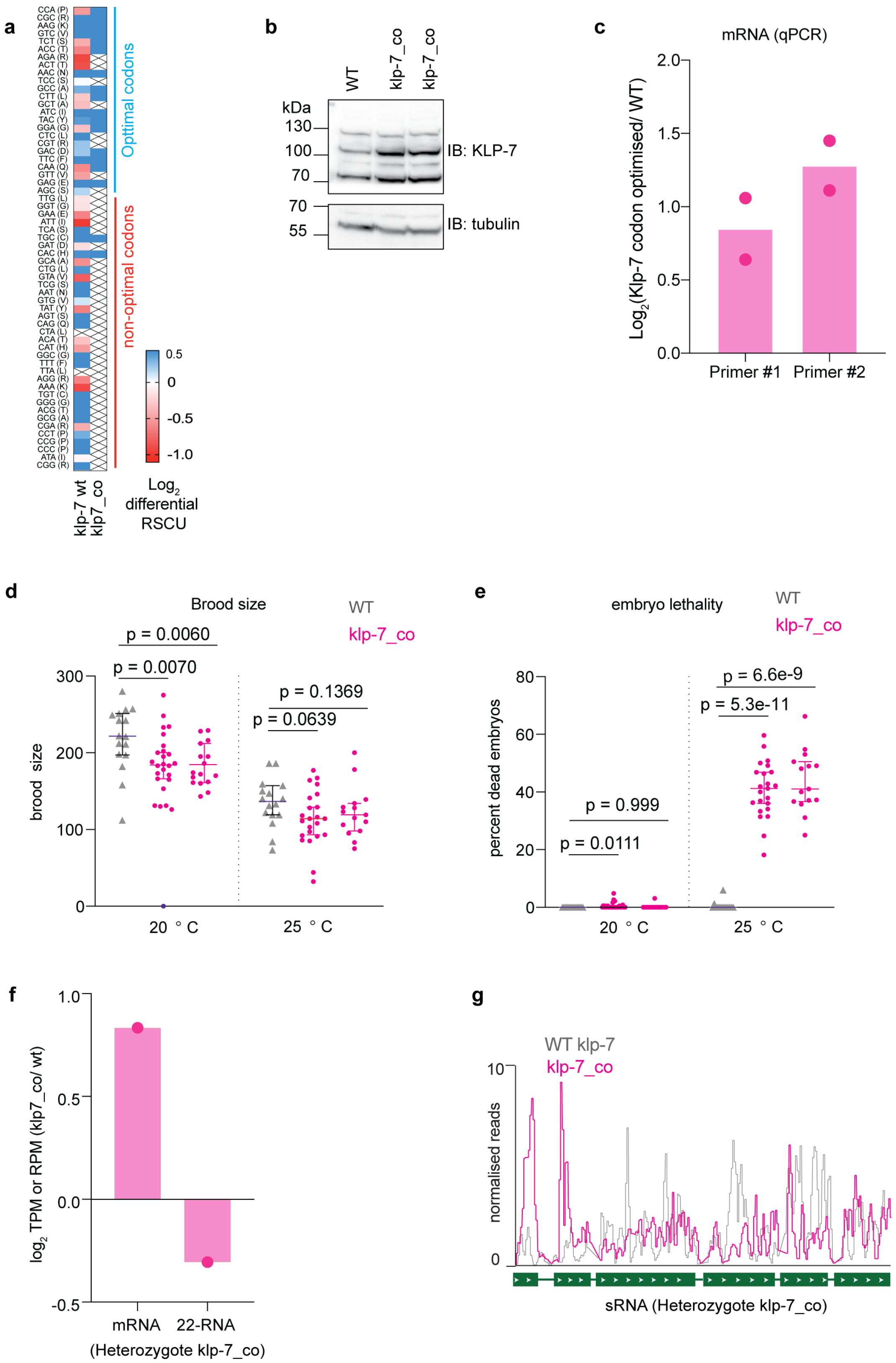

Supplementary Fig. 9: Effects of codon optimization of CSR-1 target - *klp-7*.

a, heat map showing log_2 fold change in RSCU for either WT *klp-7* or *klp-7_co* compared to genes showing neutral translational efficiency of 1 as explained in methods (similar to Fig. 5b, c). The blue line highlights optimal codons used by genes with high TE, and the red line

highlights non-optimal codons. × denotes an absence of the codon in the coding sequence. **b,** immunoblot showing expression of the KLP-7 protein in WT strain and *klp-7_co* strain where endogenous *klp-7* was replaced by modified *klp-7_co,* immunoblot for alpha-tubulin serves as the loading control. Data is representative of two experiments with two independent CRISPR-Cas9 lines for *klp-7_co*. **c,** $\log_2$ fold change in expression of *klp-7* in the *klp-7* codon-optimized strain, *klp-7_co,* compared to WT *klp-7* in WT strain by RT-qPCR for two biological replicates used for sequencing in Fig. 6. Data are represented as mean. **d,** Brood size of WT strain and two independent CRISPR-Cas9 lines where modified *klp-7_co* replaced endogenous *klp-7* at 20° and 25° C. **e,** Embryonic lethality observed in WT strain and the two independent CRISPR-Cas9 lines where modified *klp-7_co* replaced endogenous *klp-7* at 20° and 25° C. For (**d-e)** n = 15 to 26 animals per condition were used. Data are represented as mean ± s.d. Two-tailed P values were calculated using Mann–Whitney–Wilcoxon tests. **f,** Plot showing $\log_2$ fold change for normalized reads for mRNA and 22G-RNAs for the codon-optimized copy of *klp-7_co* compared to WT copy *klp-7* from heterozygote worms, n=1. **g,** A genomic view of normalized reads of 22G-RNA reads antisense to WT copy of *klp-7* and codon-optimized *klp-7_co* copy from heterozygote worms. Source data are provided as a Source Data file.
