# Supplementary material for: Translation and codon usage regulate Argonaute slicer activity to trigger small RNA biogenesis
Source: Nat Commun. 2021 Jun 9;12:3492. doi: 10.1038/s41467-021-23615-w (PMC8190271; doi:10.1038/s41467-021-23615-w)
Supplement: Supplementary file 10 — Description of Additional Supplementary Files [file 41467_2021_23615_MOESM10_ESM.docx]

Description of additional supplementary information

Title: Supplementary Data 1.

Description: Gene lists generated and used in this study

Title: Supplementary Data 2.

Description: IP-MS/MS - CSR-1 IP / control IP

Title: Supplementary Data 3.

Description: IP-MS/MS - CSR-1 ADH IP / CSR-1 WT IP

Title: Supplementary Data 4.

Description: IP-MS/MS -PRG-1 IP / control IP

Title: Supplementary Data 5.

Description: List of strains

Title: Supplementary Data 6.

Description: List of guide and repair template sequences used to generate CRISPR-Cas9 alleles

Title: Supplementary Data 7.

Description: Oligo pairs used for RT-qPCR
