# Supplementary material for: Translation and codon usage regulate Argonaute slicer activity to trigger small RNA biogenesis
Source: Nat Commun. 2021 Jun 9;12:3492. doi: 10.1038/s41467-021-23615-w (PMC8190271; doi:10.1038/s41467-021-23615-w)
Supplement: Supplementary file 11 — Reporting Summary [file 41467_2021_23615_MOESM11_ESM.pdf]

nature research

Corresponding author(s): Germano Cecere

Last updated by author(s): April 26, 2021

Reporting Summary

Nature Research wishes to improve the reproducibility of the work that we publish. This form provides structure for consistency and transparency in reporting. For further information on Nature Research policies, see our Editorial Policies and the Editorial Policy Checklist.

Statistics

For all statistical analyses, confirm that the following items are present in the figure legend, table legend, main text, or Methods section.

n/a	Confirmed	
☐	☒	The exact sample size (*n*) for each experimental group/condition, given as a discrete number and unit of measurement
☐	☒	A statement on whether measurements were taken from distinct samples or whether the same sample was measured repeatedly
☐	☒	The statistical test(s) used AND whether they are one- or two-sided<br>*Only common tests should be described solely by name; describe more complex techniques in the Methods section.*
☒	☐	A description of all covariates tested
☒	☐	A description of any assumptions or corrections, such as tests of normality and adjustment for multiple comparisons
☐	☒	A full description of the statistical parameters including central tendency (e.g. means) or other basic estimates (e.g. regression coefficient) AND variation (e.g. standard deviation) or associated estimates of uncertainty (e.g. confidence intervals)
☐	☒	For null hypothesis testing, the test statistic (e.g. *F*, *t*, *r*) with confidence intervals, effect sizes, degrees of freedom and *P* value noted<br>*Give P values as exact values whenever suitable.*
☒	☐	For Bayesian analysis, information on the choice of priors and Markov chain Monte Carlo settings
☒	☐	For hierarchical and complex designs, identification of the appropriate level for tests and full reporting of outcomes
☒	☐	Estimates of effect sizes (e.g. Cohen's *d*, Pearson's *r*), indicating how they were calculated

Our web collection on statistics for biologists contains articles on many of the points above.

Software and code

Policy information about availability of computer code

Data collection	Applied Biosystems™ QuantStudio™ 3 Real-Time PCR System was used for qPCR<br><br>Illumina NextSeq 500 or MiniSeq platforms were used for sequencing<br><br>Confocal images were taken using ZEISS LSM 700 microscope with 40X objective or 63X objective. Images were obtained using ZEISS ZEN black 2.3 SP1. Fluorescnece images were taken on Zeiss Axio Imager M2 and acquired using MetaMorph
Data analysis	RNA-seq analysis:<br>- Demultiplexing with Illumina bcl2fastq converter version v2.17.1.14<br>- Quality control with fastQC version v0.11.5<br>-<br>Allignment with HISAT2 version 2.0.4<br>- Genomic features annotation with featureCounts version 1.5.2<br>- Differential gene expression analysis with DESeq2 version 1.20.0<br><br>Small RNA-seq, Ribo-seq and GRO-seq analyses:<br>- Demultiplexing with Illumina bcl2fastq converter version v2.17.1.14<br>- Quality control with fastQC version v0.11.5<br>- 3' adaptor trimming with Cutadapt version 1.15<br>- Allignment with Bowtie2 version 2.3.4.1<br>- Metaprofile with deeptools package version 3.1.2<br>- gffutils<br>- Python 3.9

Distance mapping
-Scipy library (version 1.3.2)
- Matplotlib version 3.1.1

Images were processed using ImageJ software V2.0.0.

qPCR data were analyzed using QuantStudioTM Design and Analysis software V 2.2

tRNA analysis
- tRNAscan-SE 2.0

MS/MS data analysis
- Sequest HT through Proteome Discoverer (v.2.2)
- myProMS v.3.9
- MassChroQ v.2.2.1
- Database C. elegans (CAEEL) UP000001940 database

Enrichment analysis online tool
WormCat : http://wormcat.com/
http://nemates.org/MA/progs/overlap_stats.html

Codon usage analysis
bedtools version v2.27.1,
bedops version 2.4.35
bedGraphToBigWig version 4.

Read composition analyses
-samtools package

RSCU online tool
http://genomes.urv.es/CAIcal/

Graphpad Prism 9

For manuscripts utilizing custom algorithms or software that are central to the research but not yet described in published literature, software must be made available to editors and reviewers. We strongly encourage code deposition in a community repository (e.g. GitHub). See the Nature Research guidelines for submitting code & software for further information.

Data

Policy information about availability of data

All manuscripts must include a data availability statement. This statement should provide the following information, where applicable:

- Accession codes, unique identifiers, or web links for publicly available datasets
- A list of figures that have associated raw data
- A description of any restrictions on data availability

All sequencing data (GRO-seq, RNA-seq, and sRNA-seq from total lysate or IP experiments, Ribo-seq) are available at the Gene Expression Omnibus (GEO) under accession code GSE155077 (https://www.ncbi.nlm.nih.gov/geo/query/acc.cgi?acc=GSE155077). The MS proteomics data have been deposited to the ProteomeXchange Consortium via the PRIDE partner repository with the dataset identifier PXD012557 (https://www.ebi.ac.uk/pride/archive/projects/PXD012557) and PXD020293 (https://www.ebi.ac.uk/pride/archive/projects/PXD020293). Any other data supporting the findings and custom scripts of this study are available from the corresponding author on request. Custom code and data analysis workflows are available at https://gitlab.pasteur.fr/bli/bioinfo_utils. Source data are provided with this paper.

Field-specific reporting

Please select the one below that is the best fit for your research. If you are not sure, read the appropriate sections before making your selection.

☒ Life sciences ☐ Behavioural & social sciences ☐ Ecological, evolutionary & environmental sciences

For a reference copy of the document with all sections, see nature.com/documents/nr-reporting-summary-flat.pdf

Life sciences study design

All studies must disclose on these points even when the disclosure is negative.

Sample size	For RNA-seq and total sRNA-seq and GRO-seq and RT-qPCR atleast 1000 worms were used, for IP and Ribo-seq experiments atleast 10,000 worms were used. This sample size has been empirically evaluated to be sufficient to obtain large amount of RNAs or proteins content, sufficient for multiple applications. For imaging at least 5-10 individual germlines were imaged.<br>Sample size for the brood size experiments was determined according to our pre-tests in the lab using at least 10 worms for each strain.<br>For IP-MS/MS analysis were performed with at least 20,000 worms per samples. This sample size was chosen after empirical testing with different numbers of worms in IP-MS/MS and peptide coverage thus obtained.

Polysome profile for immunoblot was performed with at least 40,000 worms. This was evaluated based on protein content obtained post fractionation for at least performing two blots.
For IP-radiolabeling of sRNA 10,000 worms were used and the sample size was determined by testing P32-labelling efficiency of the immunoprecipitate by imaging the radiograph.

Data exclusions	Dead worms were excluded from the counting in brood size experiments.
Replication	Almost all the experiments shown in this study were performed independently at least two times and no inconsistent results were observed. All attempts at replication were successful.
Randomization	Our experiments were not randomized and controlling covariates was not necessary. For all experiments, control vs. experimental samples were treated in parallel. Samples were allocated into different groups based on genotype for all genetic/mutant analyses performed. Animals were randomly chosen from stock plates for brood size experiments.
Blinding	Plates for brood size assays were assigned with randomized labels and the investigators counting each group were blinded to each group identity.

Reporting for specific materials, systems and methods

We require information from authors about some types of materials, experimental systems and methods used in many studies. Here, indicate whether each material, system or method listed is relevant to your study. If you are not sure if a list item applies to your research, read the appropriate section before selecting a response.

Materials & experimental systems

n/a	Involved in the study	
☐	☒	Antibodies
☒	☐	Eukaryotic cell lines
☒	☐	Palaeontology and archaeology
☐	☒	Animals and other organisms
☒	☐	Human research participants
☒	☐	Clinical data
☒	☐	Dual use research of concern

Methods

n/a	Involved in the study	
☒	☐	ChIP-seq
☒	☐	Flow cytometry
☒	☐	MRI-based neuroimaging

Antibodies

Antibodies used

anti-KLP-7 , a gift from Desai lab, dilution used 1:1000
α-FLAG antibody (F3165, Sigma) dilution used 1:1000
anti-tubulin (Ab6160, Abcam) dilution used 1:1000
Anti-RPS3 (ab128995, Abcam) dilution used 1:3000
anti-GAPDH (Ab125247, Abcam) dilution used 1:2000
anti-PGL-1 (a gift from the Strome laboratory) dilution used 1:2000
anti-PRG-1 (a gift from the Mello laboratory) dilution used 1:2000
HRP conjugated anti-rabbit (31460, Pierce) dilution used 1:10000
HRP conjugated anti-mouse (31430, Pierce) dilution used 1:10000
HRP conjugated anti-rat (A9037, Sigma) dilution used 1:10000
Anti-FLAG M2 Magnetic Agarose Beads suspension (Sigma M8823)
GFP-Trap Magnetic Agarose (Chromotek gtma-10)

Validation

anti-KLP-7 antibody was validated in Gerson-Gurwitz et. al. Cell 2016 http://dx.doi.org/10.1016/j.cell.2016.02.040
anti-PGL-1 antibody was validated in Kawasaki, I. et al. PGL-1, a Predicted RNA-Binding Component of Germ Granules, Is Essential for Fertility in C. elegans. Cell 94, 635–645 (1998).
anti-PRG-1 antibody was validated in Simon, M. et al. Reduced Insulin/IGF-1 Signaling Restores Germ Cell Immortality to Caenorhabditis elegans Piwi Mutants. Cell Reports 7, 762–773 (2014).
anti-FLAG antibody (F3165, Sigma) has been validated by vendor (https://www.sigmaaldrich.com/catalog/product/sigma/f3165).
anti-tubulin (Ab6160, Abcam) has been validated by Barucci et al. Nature Cell Biology 2020.
anti-GAPDH (Ab125247, Abcam) has been validated by Barucci et al. Nature Cell Biology 2020.
HRP conjugated anti-rabbit (31460, Pierce) has been validated by Barucci et al. Nature Cell Biology 2020.
HRP conjugated anti-mouse (31430, Pierce) has been validated by Barucci et al. Nature Cell Biology 2020.
HRP conjugated anti-rat (A9037, Sigma) has been validated by Barucci et al. Nature Cell Biology 2020.
Anti-FLAG M2 Magnetic Agarose Beads suspension (Sigma M8823) has been validated by Barucci et al. Nature Cell Biology 2020.
GFP-Trap Magnetic Agarose (Chromotek gtma-10) has been validated by Barucci et al. Nature Cell Biology 2020.
Anti-RPS3 (ab128995, Abcam) has been validated in this study by immunoblot.

Animals and other organisms

Policy information about studies involving animals; ARRIVE guidelines recommended for reporting animal research

Laboratory animals	All the data collected for this study derived from hermaphrodite Caenorhabditis elegans nematode culture at Late L4 or adult stages

Laboratory animals	as described in methods<br>List and description of strains is present in Supplementary Data 5
Wild animals	No wild animals has been used in this study
Field-collected samples	This study did not involve samples collected from the field.
Ethics oversight	No ethical approval was required.

Note that full information on the approval of the study protocol must also be provided in the manuscript.
